# Domestic

**Published:** 1841-08-28

**Authors:** 


### MEDICAL EXAMINER.

DEVOTED TO MEDICINE, SURGERY, AND THE COLLATERAL SCIENCES.

No. 35.] PHILADELPHIA, SATURDAY, AUGUST 28, 1841. [Vol. IV.

### DOMESTIC.

*Case of Disunited Fracture successfully treated with the Seton.* By William H. Donne, M. D. Reported by A. Martin, resident student in the Louisville Marine Hospital.—William Wheeling, a labourer, a native of Ireland, aged twenty-three years, of intemperate habits, was admitted into the Louisville Marine Hospital on the 14th June, 1840, with a transverse fracture of the tibia about three inches below the femoro-tibial articulation, caused by falling into the hold of a steamboat and striking the limb against a bar of iron. There was no apparent displacement of the fragments, and very little tumefaction of the soft parts was observed in the vicinity of the fracture. On moving the limb in various directions, crepitation was distinctly audible, and the superior fragment could be made slightly prominent by flexing the knee. No fracture of the fibula, however, could be detected upon the most careful examination. The roller was firmly applied by Dr. T. L. Caldwell, then in attendance, from the toes upwards above the point of fracture, with injunctions to moisten it as often as necessary with aqua ammoniæ and diluted alcohol. After the tumefaction had subsided, two splints, composed of book-binder's board, previously soaked in warm water, were applied and moulded to the limb by the roller, which was carried above the point of fracture as in the first dressing. The limb was now placed on an inclined plane, with directions to keep the dressings immediately over the fracture moistened from time to time. This treatment was continued for six weeks, when the splints, &c. were removed. Upon examination it was ascertained that no union had taken place. All dressings were then discontinued, and the patient permitted to take exercise on crutches through the ward, for the space of three weeks without improvement. Friction, and rubbing the fragments together by moving the limb in different directions, were likewise resorted to without any beneficial results. On the 21st of August, with the concurrence and assistance of Dr. T. L. Caldwell, (whose tour of attendance had expired a few weeks previously,) Dr. Donne introduced a seton between the fragments, by first dividing the integuments and aponeuroses in the anterior tibial region to a small extent. The tibialis anticus muscle presenting itself, a few of its fibres were detached from their connection with the outer surface of the tibia, to facilitate the passage of the needle. The hæmorrhage was comparatively slight, nor did the patient complain of much pain during the operation.

Five days elapsed before there was any appearance of inflammation, which increased gradually until the 27th, when the wound made by the egress of the needle began to suppurate.

29th. Suppuration in both wounds; patient complains of a stinging pain in the vicinity of the fracture.

31st. Pain more severe; inflammation much diffused.

Sept. 2d. Complains of great pain; local inflammation increased.

4th. Scultetus' bandage applied, moistened with ammoniated lotion.

7th. Inflammation still extending; some febrile action; tongue furred; restless through the night; bowels open without medicine. Seton removed; a considerable quantity of cream coloured pus with some coagula discharged from the internal aperture.

10th. Internal wound continues to discharge healthy matter.

12th. The quantity of matter diminished; a compress with an aperture in its centre, and Scultetus' bandage applied.

14th. Discharge much diminished; roller applied.

17th. No pain in the limb; use splints and roller.

20th. Wound closed; ordered porter and generous diet.

25th. Severe pain in the fractured limb; restless during the night.

26th. Bandages and splints removed; inflammation of the integuments and subcutaneous cellular tissue in the vicinity of the internal wound; limb much swollen; fluctuation evident, about eight ounces of sanguineo-purulent matter discharged.

27th. Matter again collected; counter opening made—affording a free discharge.

Oct. 1st. Discharge very slight; pain greatly diminished by incision.

4th. Health improving; discharge ceased; counter opening closed.

8th. Free from pain; limb seems firm; no appreciable motion in the site of the fracture.

12th. Tumefaction of the limb diminished; patient exercises on crutches, and can support more weight on the leg than at any previous time since the accident.

16th. Patient walks about the ward with a cane; has a good appetite, and rests well at night.

20th. Bandages dispensed with; limb free from pain and daily gaining strength.

28th. Patient can bear his whole weight on the limb; walks well without any inconvenience.

A few weeks afterwards he was discharged cured.

There seems to be much discrepancy of opinion relative to the length of time that the seton should be retained in cases of disunited fracture, Dr. Gibson and others insisting on its retention for months, or until the deposition of ossific matter shall have commenced, while Mr. Liston, who is equally orthodox, thinks one or two weeks sufficient.

Nothing definite, we imagine, can be laid down as to the time which may be required to induce ossific deposition by the seton, for not only are constitutional peculiarities to be taken into consideration, but the location of the injury, and many adventitious circumstances, must influence the recuperative efforts of nature.—*Western Journal of Medicine and Surgery.*

---

*A Case of Hydrocele*, reported to the Medical Society of Tennessee at its meeting in May. By Ferdinand Stith, M. D., of Franklin, Tenn.—A young gentleman from the country, aged about twenty-five years, of fair skin, light hair, and blue eyes, called on me, on the 17th of March, 1836, to have himself relieved of a disease of the testis. On examination, I found him the subject of hydrocele, of two or three years standing. I explained to him the nature of his case, and gave him to understand what sort of an operation he would have to submit to, and ho w long he would probably be confined.

He concluded to undergo the operation at once, and accordingly took lodgings in town for that purpose. He manifested great solicitude to keep the nature of his disease secret, and desired me, if I could, to dispense with the aid of an assistant, to which I readily consented; and having made the necessary preparations, I repaired to his room for the purpose of operating.

Having placed him in a favourable position on the edge of the bed, and making myself acquainted with the position of the testis, I laid open the sac with my scalpel to the extent of an inch and a half. So soon as the encysted fluid, amounting to about a pint, had escaped, I introduced my finger into the tunica vaginalis testis, for the purpose of bringing its surface, and the surface of the testis, into view, in order that I might the more correctly judge of the condition of the parts. I found the surface generally presenting a normal appearance, all but a portion directly over the testis, on which there was a slight roughness to the touch, the sensibility of which was so exquisite, with the excessive nervousness of the patient, that when I attempted to introduce a fold of the softest lint, I found him falling into a syncope, and was compelled to desist.

After giving him some stimuli and waiting a few minutes, I made a second attempt to introduce a fold of lint, made still less irritating by covering it with simple cerate. This time I succeeded partially, but the operation gave him great pain. I passed a small strip of lint above the testis, in the direction of the spermatic cord. I had made up my mind to effect a radical cure, and had so promised my patient; but at this stage of the business I began to feel in some doubt as to the best mode of proceeding. I was afraid of the effects of stimulating injections on a surface that would not bear the slightest touch, but could not think of suffering the incision to heal without obliterating the cavity of the sac. And it now occurred to me, that the least irritating article that could be devised, would be a piece of the softest kid skin, such as I use for animal ligatures, saturated with the mucilage of gum arabic and white sugar. This I succeeded in introducing with a probe, without giving the slightest pain.

The strips of lint and of kid skin having now been introduced, in different directions, I proceeded to close the wound, leaving the ends of the strips slightly projecting from its lips, and applied a suspensory bandage.

In the course of two or three days the adhesive inflammation was fully established—indeed, was something more than I had desired, and I had to bleed him once, and give him two or three gentle purgatives. In the course of thirty-six or forty-eight hours from the time of the operation, the kid skin had become fixed in its position by the adhesive inflammation, and on the sixth day the projecting extremities dropped off, that portion of the sac in which it lay having contracted and firmly united. While this was the case with one portion of the sac, that which was occupied by the lint had not suppuratad sufficiently to allow me to withdraw it until the ninth day.

On the twelfth day, all the external openings had closed, and I put the patient on the use of camphorated mercurial ointment to hasten the reduction of the parts to their natural condition. He was now able to leave his room, had begun to walk about town, and after a few days more, returned in good health to the country. He has never been threatened with a return of the symptoms, and is now, as he has been at all times since he recovered from the operation, in the enjoyment of fine health.

My object in reporting this case to the society is two-fold; first, to direct the attention of surgeons to the use of the kid skin in such cases, as giving less pain, and being more likely to answer every indication than any agent hitherto employed;—and, secondly, to suggest the idea of attempting the radical cure of hernia, by, in the first place, reducing it, then opening the sac, and, lastly, introducing the kid skin. Of course, for some days, the patient, after such an operation, would be obliged to maintain the horizontal posture, to use compresses so adjusted as to prevent the intestine from falling into the passage, and to keep the parts in a state of absolute rest. The union, in my opinion, would be effected in forty-eight hours; and if the patient were in a favourable state for the operation, and received judicious treatment afterwards, I should not apprehend any danger of an extension of the inflammation beyond the limits of the enclosed kid skin.
*Ibid.*

---

*New Cure for Hydrocele.*—Colonel W. M., a gentleman of Smith county, Tenn., eminent for his public and private worth, aged about 75 years, had been the subject of hydrocele during nearly a third part of that time, and although the collection of fluid had been slow, the tumour had attained a very uncomfortable size before he was relieved of it by an operation which, in this connexion, may be termed novel. In attempting to mount his horse, early in May last, his saddle turned, and he fell on the other side upon his shoulders, by which his body received a smart concussion but no injury. He rose without difficulty, and proceeded to the place of religious worship where he was going, feeling only some slight pain and a sense of tension in the tumour along the course of the spermatic cord. A few nights after the fall he thought that he found less trouble than usual in disposing of the tumour preparatory to sleeping, but it did not occur to him that this was owing to its being diminished in size, nor was his attention called to this circumstance until a week after the accident, when on retiring to bed he was surprised at finding that it had entirely disappeared. In one week the collection of fluid, amounting, it is supposed, to a quart, was taken up by absorption, in consequence, it would seem, of a new action in the parts set up by the shock given by the fall.

It has now been upwards of five weeks since the Colonel was thus most unexpectedly relieved of his infirmity, and there is yet no appearance of its return.

A circumstance followed the termination of this case which the physicians who attended the Colonel believed had some connexion with the absorption of the fluid. He was attacked, seven days after the tumour vanished, with profuse diarrhœa, his discharges resembling the rice water passages in cholera. He was greatly prostrated by the disease, one of the most distressing symptoms of which was an uncontrollable propensity to sleep. He recovered gradually, and now enjoys excellent health. He is a man of strict temperance and regular habits, and is not conscious of having brought on the attack of diarrhœa by any indiscretion. His case is a curious one as to the mode in which he was relieved of the hydropic effusion, and not less so, if it be admitted, that the discharge of the absorbed fluid from the system through the bowels was the cause of the diarrhœa, which, we confess, we are not quite prepared to do.—*Western Journal of Medicine and Surgery.*

